# Screening Fungal Endophytes Derived from Under-Explored Egyptian Marine Habitats for Antimicrobial and Antioxidant Properties in Factionalised Textiles

**DOI:** 10.3390/microorganisms8101617

**Published:** 2020-10-21

**Authors:** Ahmed A. Hamed, Sylvia Soldatou, M. Mallique Qader, Subha Arjunan, Kevin Jace Miranda, Federica Casolari, Coralie Pavesi, Oluwatofunmilay A. Diyaolu, Bathini Thissera, Manal Eshelli, Lassaad Belbahri, Lenka Luptakova, Nabil A. Ibrahim, Mohamed S. Abdel-Aziz, Basma M. Eid, Mosad A. Ghareeb, Mostafa E. Rateb, Rainer Ebel

**Affiliations:** 1 Microbial Chemistry Department, National Research Centre, 33 El-Buhouth Street, Dokki, Giza 12622, Egypt; ahmedshalbio@gmail.com (A.A.H.); mohabomerna@yahoo.ca (M.S.A.-A.); 2 Marine Biodiscovery Centre, Department of Chemistry, University of Aberdeen, Aberdeen AB24 3UE, UK; s.soldatou@rgu.ac.uk (S.S.); r02sa17@abdn.ac.uk (S.A.); r02kjm17@abdn.ac.uk (K.J.M.); f.casolari.19@abdn.ac.uk (F.C.); coralie.pavesi@edu.mnhn.fr (C.P.); r01oad17@abdn.ac.uk (O.A.D.); 3 School of Computing, Engineering & Physical Sciences, University of the West of Scotland, Paisley PA1 2BE, UK; mallique.qader@gmail.com (M.M.Q.); bathini.thissera@uws.ac.uk (B.T.); m.eshelli@hotmail.com (M.E.); 4 National Institute of Fundamental Studies, Hantana Road, Kandy 20000, Sri Lanka; 5 College of Pharmacy, Adamson University, 900 San Marcelino Street, Manila 1000, Philippines; 6 Food Science & Technology Department, Faculty of Agriculture, University of Tripoli, Tripoli 13538, Libya; 7 Laboratory of Soil Biology, University of Neuchatel, 2000 Neuchatel, Switzerland; lassaad.belbahri@unine.ch; 8 Department of Biology and Genetics, Institute of Biology, Zoology and Radiobiology, University of Veterinary Medicine and Pharmacy, 04181 Kosice, Slovakia; lenka.luptakova@uvlf.sk; 9 Textile Research Division, National Research Centre, Scopus Affiliation ID 60014618, 33 EL Buhouth St., Dokki, Giza 12622, Egypt; nabibrahim49@yahoo.co.uk (N.A.I.); basmaeid@yahoo.com (B.M.E.); 10 Medicinal Chemistry Department, Theodor Bilharz Research Institute, Kornaish El Nile, Warrak El-Hadar, Imbaba, Giza 12411, Egypt; mosad_tbri@hotmail.com

**Keywords:** endophytic fungi, antimicrobial, antioxidant, GNPS, textiles

## Abstract

Marine endophytic fungi from under-explored locations are a promising source for the discovery of new bioactivities. Different endophytic fungi were isolated from plants and marine organisms collected from Wadi El-Natrun saline lakes and the Red Sea near Hurghada, Egypt. The isolated strains were grown on three different media, and their ethyl acetate crude extracts were evaluated for their antimicrobial activity against a panel of pathogenic bacteria and fungi as well as their antioxidant properties. Results showed that most of the 32 fungal isolates initially obtained possessed antimicrobial and antioxidant activities. The most potent antimicrobial extracts were applied to three different cellulose containing fabrics to add new multifunctional properties such as ultraviolet protection and antimicrobial functionality. For textile safety, the toxicity profile of the selected fungal extract was evaluated on human fibroblasts. The 21 strains displaying bioactivity were identified on molecular basis and selected for chemical screening and dereplication, which was carried out by analysis of the MS/MS data using the Global Natural Products Social Molecular Networking (GNPS) platform. The obtained molecular network revealed molecular families of compounds commonly produced by fungal strains, and in combination with manual dereplication, further previously reported metabolites were identified as well as potentially new derivatives.

## 1. Introduction

The emergence of antimicrobial resistance (AMR) is one of the current global health challenges, and it refers to the ability of microorganisms to stop the action of antimicrobial agents, thereby increasing the prevalence and the associated risks of infections by pathogenic bacteria, fungi, viruses, and parasites [1]. Several reports highlighted the potential dangers of AMR to global public health and economics. One of these reports estimated that, if the current rate of continuous increase of AMR should prevail, 300 million people are expected to die prematurely over the next 35 years [2]. This report also predicted that, by 2050, the world will lose between 60 and 100 trillion USD if no action is taken toward AMR. In developing countries such as Egypt, the continuous misuse and overuse of antibiotics without prescription and medical supervision play a significant role in the development of antimicrobial resistance, especially in hospital-acquired infections [3]. Recent reports showed that textile can play a significant role in reducing and preventing nosocomial infections. Textiles under appropriate temperature and moisture conditions are an excellent substrate for microbial growth. These contaminated textiles act as a rich source for microbial transmission to susceptible patients. Studies hypothesize that the use of antimicrobial textiles may significantly reduce the risk of nosocomial infections [4].

Despite the undeniable achievements and success of chemical synthesis of antimicrobial compounds, nature is still considered a treasure trove and a highly attractive renewable source of a structurally diverse array of natural products that have served as lead structures for novel drug development and continue to do so [5]. There is an urgent need to discover new drug candidates with novel mechanisms of action to counter the continuous increase of AMR [1]. Naturally occurring bioactive compounds derived from endophytic fungal extracts can attenuate these harmful effects [6]. Endophytic fungi are considered a vital source of bioactive secondary metabolites with numerous biological applications [7,8,9]. Many endophytic fungal strains from different environmental and geographical areas were isolated and screened to assess their ability to produce novel molecules belonging to various compounds classes, including macrolides, terpenoids, alkaloids, or peptides, and displaying antimicrobial, antioxidant, antiviral and anticancer activity [10,11,12,13,14,15].

As the number of natural products reported in the literature is steadily increasing, dereplication strategies are essential in early stages of the fractionation-guided process to avoid isolating known metabolites. The Global Natural Products Social Molecular Networking (GNPS) is a platform which allows rapid and automated comparison of fragmentation patterns based on high-resolution MS/MS data that leads to effective dereplication [16]. GNPS clusters compounds with similar structural features which translate into similar fragmentation patterns into groups of molecular families. A molecular network consists of nodes which correspond to parent ions and are linked into groups with edges which represent a cosine similarity score. Molecular networking provides effective and rapid dereplication of large and complex MS/MS datasets and has been successfully implemented in natural product research to accelerate targeted isolation of potentially new metabolites [17,18,19].

In late 2017, we initiated a collaborative project between Egypt and the UK aiming at the isolation of new endophytic fungal strains from under-explored marine habitats in different locations in Egypt to be screened for their antimicrobial effects, with the ultimate aim of incorporating their bioactive extracts or metabolites in textiles used in Egyptian hospitals to reduce nosocomial infections. In our efforts to assess the biological activities and the chemical diversity of such fungal strains, we isolated 32 fungal strains from marine organisms and plants collected from Hurghada, Red Sea, and Wadi El-Natrun. Based on initial biological assays and LC/MS analysis of their crude extracts, 21 fungal isolates were prioritised for further analysis and identified using a combination of 18S rRNA, ITS rRNA, β-tubulin, and calmodulin gene sequencing. The most potent antimicrobial extracts were selected for the functionalization of cellulose containing fabrics to produce textile with UV-protection and antimicrobial functional properties. Moreover, chemical investigation of the most active extracts was carried out by the analysis of MS/MS data obtained from their crude extracts through the GNPS platform, and the results revealed several already reported fungal natural products which clustered with unidentified parent ions, suggesting the presence of potentially new secondary metabolites.

## 2. Materials and Methods

### 2.1. Sample Collection

Plant and marine samples were collected from two different locations in Egypt, Wadi El-Natrun depression (El-Beheira Governorate) and Hurghada (Red Sea Governorate). In total, 10 plant samples were collected from two saline lakes of Wadi El-Natrun (Al-Hamra and Al-Beida), and 9 marine samples (6 sponges, 1 soft coral, 1 sea grass, and 1 alga) were collected using SCUBA from two reefs in Hurghada, site (1) Abu Monqar island at N 27°12′53.7″, E 33°51′11.15″, and site (2) Makady Bay South at N 26°59′42.87″, E 33°54′4.02″, at a depth between 5 and 10 m. Plant and marine samples were transferred in ice boxes to the Microbial Chemistry Department, National Research Centre (NRC), Egypt, where each specimen was given a unique code, photographed, and stored at 4 °C and −20 °C, respectively, and was kept for further investigation and analysis.

### 2.2. Isolation of Endophytic Fungi

Isolation of endophytic fungi from plants and marine organisms was carried out using two different media: malt agar (MA; malt extract 15 g, sea salt 24.4 g, agar 20 g, distilled water up to 1 L, pH 6) and potato dextrose agar (PDA; potato extract 4 g, dextrose 20 g, sea salt 24.4 g, agar 20 g, distilled water up to 1 L, pH 6). For plants, healthy leaves were washed thoroughly with tap water and surface sterilised in 70% ethanol for 1 min, followed by subsecutive rinsing in sterile distilled water for 1 min, 2% sodium hypochlorite for 1 min, and finally 3 times in sterile distilled water. Sterilised leaves were dried under sterile conditions, and small segments were incubated on PDA supplemented with filter-sterilised nalidixic acid (50 mg/L) and chloramphenicol (200 mg/L) to suppress the growth of bacteria. For marine sponges and algae, approximately 1 cm^3^ of the inner tissue of each sample was excised under sterile conditions and directly placed onto MA or PDA following a previously reported protocol [20]. The plates were incubated at 28 °C until growth of endophytic fungi was observed. Individual colonies were picked and repeatedly sub-cultured until pure colonies were obtained. The pure fungal isolates were preserved as glycerol stocks and stored at −20 °C in the Microbial Chemistry Department, National Research Centre (NRC), Egypt.

### 2.3. Production and Preparation of Fungal Extracts

Fungal isolates were cultivated on rice media (100 g commercial rice in 100 mL artificial sea water, adjusted to 50% natural salinity). The cultures were incubated for 15 days at 28 °C under static conditions. After incubation, the cultures were extracted with ethyl acetate (EtOAc), and the organic phases were dried under vacuum to obtain the crude extracts.

### 2.4. DNA Extraction, Amplification and Sequencing

The molecular identification of selected fungal strains was carried out by extraction of the genomic DNA using Qiagen DNeasy Mini Kit following the manufacturer’s instructions. The PCR reaction mixture was as follows: 1 µg genomic DNA, 1 µL (20 µM of each primer), 10 mM dNTPs mixture, 2 units of Taq DNA polymerase enzyme, and 10 µL 5× reaction buffer. Initially, amplification reactions for 18S and ITS genes, respectively, were performed using 3 primer pairs; NS3 (5′-GCAAGTCTGGTGCCAGCAGCC-3′)/NS4 (5′-CTTCCGTCAATTCCTTTAAG-3′), NS1 (5′-GTAGTCATATGCTTGTCTC-3′)/NS8 (5′-TCCGCAGGTTCACCTACGGA-3′), and ITS1 (5′-TCCGTAGGTGAACCTGCG-3′)/ITS4 (5′-TCCTCCGCTTATTGATATGC-3′ [21], and the following PCR thermal profile: denaturation step at 94 °C for 5 min, followed by 35 cycles of 94 °C for 30 s, 55 °C for 30 s, 72 °C for 90 s, and a final extension step at 72 °C for 5 min. For amplification of -tubulin genes, primer pair Bt2a (5′-TTCCCCCGTCTCCACTTCTTCATG-3′)/Bt2b (5-GACGAGATCGTTCATGTTGAACTC-3′) was used with the following PCR thermal profile: denaturation step at 95 °C for 5 min, followed by 35 cycles of 95 °C for 30 s, 58 °C for 30 s, 72 °C for 60 s, and a final extension step at 72 °C for 7 min [22]. For amplification of CaM genes, primer pair cmd5 (5′-CCGAGTACAAGGAGGCCTTC-3′)/cmd6 (5-CCGATAGAGGTCATAACGTGG-3′) was used with the following PCR thermal profile: denaturation step at 95 °C for 10 min, followed by 35 cycles of 95 °C for 30 s, 55 °C for 30 s, 72 °C for 60 s, and a final extension step at 72 °C for 7 min [23]. The amplified products were subjected to agarose gel electrophoresis, and bands of the expected sizes were excised and purified using Montage PCR Clean up kit (Millipore) or JeneJET purification kit (ThermoFisher Scientific, Basel, Switzerland) and shipped for sequencing by two commercial services, SolGent and Macrogen, South Korea. The resulting sequences were analysed by BLASTN to study their similarity and homology with the respective target gene sequences contained in the NCBI database. The phylogenetic tree was constructed based on the maximum-likelihood (ML) algorithm [24] using MEGA6 [25], with evolutionary distances computed using the Kimura 2-parameter model [26]. Validity of branches in the resulting trees was evaluated by bootstrap resampling support of the data sets with 1000 replications. Isolate 13A was not included, as only ITS sequence information was available, while strain M13 did not provide DNA of sufficient quality during the second round of sequencing.

### 2.5. Antimicrobial Activity

Antimicrobial screening of the fungal crude extracts was carried out against a set of test microbes comprising the penicillin-resistant Gram-positive bacterium *Staphylococcus aureus* ATCC 6538-P, the Gram-negative bacterium *Pseudomonas aeruginosa* ATCC 27853, the yeast *Candida albicans* ATCC 10231, and the fungus *Aspergillus niger* NRRLA-326. Initial screening and selection of strains for further chemical analysis was based on the agar diffusion method [27] (data not shown). For active fungal extracts, minimal inhibitory concentrations (MIC) were determined using the microplate dilution method [28]. In total, 10 μL of extracts at different concentrations were added to 180 μL of culture medium, i.e., lysogeny broth for bacteria or potato dextrose broth for fungi, followed by addition of 10 μL of bacterial or fungal suspension at the log phase. The plates were incubated overnight at 37 °C, and the absorbance was measured at OD600 using a Spectrostar Nano Microplate Reader (BMG Labtech GmbH, Allmendgrun, Germany). Initially, eight serial dilutions (250, 125, 62.5, 31.25, 15.63, 7.81, 3.90, 1.95 µg/mL) were prepared, and, based on the results obtained, further dilutions bracketing the lowest concentration with no observable growth of bacteria or fungi were tested in steps of 1 µg/mL. MICs are reported as the average of the lowest concentrations with no observable growth of bacteria or fungi determined in three independent experiments. Ciprofloxacin and nystatin were used as positive controls.

### 2.6. Total Antioxidant Capacity (TAC)

The total antioxidant capacity (TAC) of each fungal extract was determined following the phosphomolybdenum method [29] using ascorbic acid as standard. This assay is based on formation of a green coloured Mo(V) phosphate complex if analytes present in the sample are capable of reducing Mo(VI) to Mo(V). In total, 0.5 mL of each extract (at a concentration of 100 µg /mL in methanol) was combined in dried vials with 5 mL of reagent solution (0.6 M sulfuric acid, 28 mM sodium phosphate and 4 mM ammonium molybdate). The reaction mixture was incubated in a thermal block at 95 °C for 90 min. After the samples had cooled at room temperature, the absorbance was measured at 695 nm against a blank consisting of all reagents and solvents without the sample, which was incubated under the same conditions. All experiments were carried out in triplicate. The antioxidant activity of the sample was expressed as the number of equivalents of ascorbic acid (AAE), and the total antioxidant capacity (TAC) was calculated as follows:

$$TAC\;=\;\left(\frac{Absorbance\;sample}{Absorbance\;ascorbic\;acid}\right)*1000$$

### 2.7. Grafting of MCT-βCD onto Cellulosic Substrates

Pre-chemical modification of the different cellulosic substrates (size 3 × 6 cm), namely knitted cotton jersey (30/1) (100g/m^2^), knitted viscose jersey (30/1) (100 g/m^2^) and plain (1/1) woven cotton (120 g/m^2^), was carried out using the pad-dry-cure method. Cellulosic substrates were immersed for 20 min with 25 g/L MCT-βCD and 8 g/L Na_2_CO_3_ as a catalyst, followed by padding to a wet pick-up at 80% wetness, drying at 100 °C for 3 min, and curing at 160 °C for 3 min. Before testing, a final washing step was used to remove excess, partially hydrolysed and unfixed reactant, followed by drying.

### 2.8. Post-Hosting of Bio-Active Extracts into Hydrophobic Cavities to Impart the Demanded Functionalities

Portions of pre-modified cellulosic substrates containing the hosting cavities were post-loaded with the selected active extracts, then suspended in 15 mL methanol by exhaustion technique using an IR-dyeing machine at 40 °C for 1 h, followed by thoroughly rinsing and air drying.

### 2.9. Methods of Analysis

Nitrogen content of the pre-modified cellulosic substrates was assessed by Kjeldhal method. Antimicrobial effects of bioactive extract-loaded substrates against *S. aureus*, *E. coli*, *C. albicans*, and *A. niger* were evaluated qualitatively according to a published method [27]. The UV protection factor (UPF) of untreated, pre-modified, and functionalized fabric samples was evaluated according to the Australian/New Zealand Standard (AS/NZS 4399/1996) and ranked as follows: good (UPF:15–24), very good (UPF: 25–39), and excellent (UPF > 40).

### 2.10. In Vitro Cytotoxicity

Cytotoxicity against normal human diploid fibroblasts (WI-38) was tested using the MTT assay [30]. The cell lines were obtained from American Type Culture Collection (ATCC) via Holding company for biological products and vaccines (VACSERA), Cairo, Egypt.

### 2.11. Mass Spectral Data Acquisition

Extracts were dissolved in methanol at a final concentration of 0.1 mg/mL, centrifuged, and injected onto a Bruker MAXIS II Q-ToF mass spectrometer coupled to an Agilent 1290 UHPLC system. Separation was achieved using a Phenomenex Kinetex XB-C18 (2.6 mM, 100 × 2.1 mm) column and the following LC gradient profile: 5% MeCN + 0.1% formic acid to 100% MeCN + 0.1% formic acid in 15 min. MS parameters were: mass range *m*/*z* 100–2000, capillary voltage 4.5 kV, nebulizer gas 5.0 bar, dry gas 12.0 L/min, and dry temperature of 220 °C. MS/MS experiments were conducted under Auto MS/MS scan mode with no step collision.

### 2.12. Molecular Networking

The MS/MS data obtained for 21 bioactive fungal isolates were converted from Bruker DataAnalysis (.d) to .mzXML file format using MSConvert (Available online: http://proteowizard.sourceforge.net/index.html). A molecular network was created using the online workflow at GNPS (Available online: https://gnps.ucsd.edu/). The data were filtered by removing all MS/MS peaks within ±17 Da of the precursor *m*/*z*. MS/MS spectra were window-filtered by choosing only the top 6 peaks in the ± 50 Da window throughout the spectrum. The data were clustered with MS-Cluster with a parent mass tolerance of ±2 Da and a MS/MS fragment ion tolerance of ±0.5 Da to create consensus spectra. Further, consensus spectra that contained less than 1 spectrum were discarded. A network was created where edges were filtered to have a cosine score above 0.5 and more than 6 matched peaks. Further edges between two nodes were kept in the network only if each of the nodes appeared in each other’s respective top 10 most similar nodes. The spectra in the network were then searched against GNPS’ spectral libraries. The library spectra were filtered in the same manner as the input data. All matches kept between network spectra and library spectra were required to have a score above 0.6 and at least 6 matched peaks. Cytoscape version 3.6.1 was used to visually display the data as a network of nodes and edges [31].

## 3. Results

### 3.1. Fungal Isolation and Cultivation

A total of 32 pure fungal strains were isolated, out of which 18 were isolated from marine samples of Hurghada, and 14 were obtained from plant samples of Wadi El-Natrun. Small scale fermentation of the isolated fungal strains was carried out on solid rice medium, and the resulting extracts were subjected to biological screening for anti-bacterial, anti-fungal, and antioxidant activities.

### 3.2. Molecular Identification

Based on a combination of biological screening results as well as LC/MS analysis, 21 fungal isolates were selected and genetically identified by extraction and sequencing of DNA at SolGent and Macrogen Companies, South Korea. The resulting sequences were aligned with closely related known sequences in GenBank. Based on their ITS and 18S rDNA sequences, isolates were identified and found to belong to eight different genera, i.e., *Alternaria*, *Aspergillus*, *Byssochlamys*, *Cladosporium*, *Epicoccum*, *Penicillium*, *Sarocladium*, and *Talaromyces*. As only isolate 13A could be identified as *Alternaria alternata* with this approach, the remaining isolates were further assessed by phylogenetic analysis of concatenated ITS rDNA, β-tubulin, and calmodulin regions, respectively (for details including GenBank accession numbers, see Table 1).

Our molecular analysis revealed interesting strains that have not been studied earlier. For example, initial DNA analysis allowed us to ascertain that AS14 fungal isolate matches the species *Epicoccum nigrum*. Given that *Epicoccum nigrum* is known to englobe two genotypes, we opted for phylogenetic analysis using type strain CBS 161.73 and other *Epicoccum nigrum* characterised by Favaro et al. [20]. Phylogenetic analysis using either ITS rDNA and β-tubulin markers separately or concatenated allowed us to unambiguously identify AS14 fungal isolate as *Epicoccum nigrum* group 2 designated by Favaro et al. [20] as *Epicoccum* sp., a new species awaiting formal description. This putative new species is understudied chemically and biologically.

### 3.3. Biological Screening

#### 3.3.1. Antimicrobial Activity

The antimicrobial results showed that some fungal extracts exhibited promising antimicrobial activity against some tested microorganisms (Table 2). Among tested extracts, M113, M13, 7S4, 7S5 and 7S9 showed significant antibacterial activity against *Staphylococcus aureus* with MIC values between 12.3 and 31.25 µg/mL, followed by M2S3, M2S4, M35, M42, 2S4, 2S6, 5S1, 7S1, 7S6, 9AS1, SHP18, and 13A with moderate activity, i.e., MIC values between 43.0 and 117.7 µg/mL. Additionally, M113, M13, 7S4, and 7S5 extracts displayed significant antibacterial activity against *Pseudomonas aeruginosa* with MIC values between 13.7 and 31.25 µg/mL. Moreover, all extracts were screened for their antifungal activity towards *Candida albicans* and *Aspergillus niger*. The extracts of the fungal isolates M113, M13, M42, 2S4, 5S1, 7S1, 7S4, 7S5, and SHP3 showed moderate inhibitory activity against *C. albicans* with MIC values between 35.0 and 125 µg/mL, whereas none of the extracts showed significant activity toward *A. niger* (Table 2).

#### 3.3.2. Total Antioxidant Capacity (TAC)

**Figure 1:**
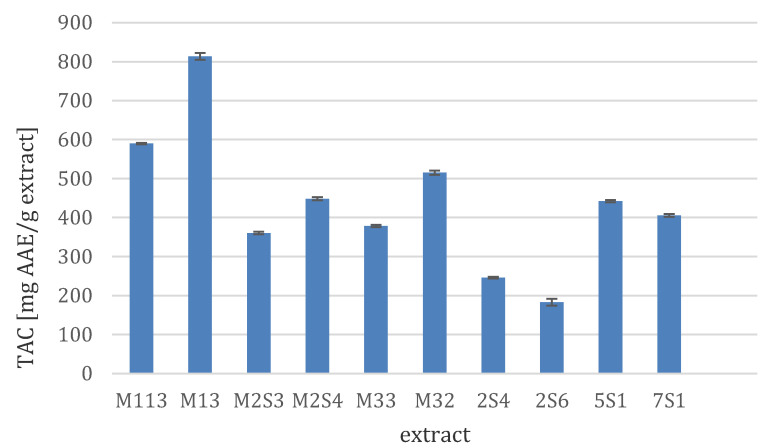
Total antioxidant capacity (TAC) of selected fungal extracts. Results are presented as means ± SD, *n* = 3) and are expressed as mg ascorbic acid equivalent (AAE)/g extract.

The fungal crude extracts were evaluated for their total antioxidant capacity (TAC) using the phosphomolybdenum method. The results presented in Figure 1 revealed that the fungal extract M13 showed the most potent antioxidant activity with a TAC value of 813.5 mg AAE/g extract (mg of ascorbic acid equivalents/g). Two extracts (M113 and M32) exhibited TAC values between 500 and 600 mg AAE/g extract, while the activity of a further three (M2S4, 5S1, and 7S1) ranged between 400 and 500 mg AAE/g extract. Four fungal extracts showed TAC values ranging between 150 and 400 mg AAE/g extract, while the remaining extracts showed weak or no activity.

Overproduction of reactive species such as reactive oxygen species (ROS) and reactive nitrogen species (RNS) leads to generation of a phenomenon known as oxidative stress. Oxidative stress represents the imbalance between the production rate of free radicals and the antioxidants in the human body, which may be followed by several health disorders, including cancer, cardiovascular diseases, inflammation, and Alzheimer’s disease [32,33]. Previous studies revealed a positive linear correlation between the antioxidant activities of the tested endophytic fungal extracts and the presence of certain chemical classes in these extracts, including phenolic acid and quinone derivatives [34], indole derivatives [6], coumarins [35], and butyrolactones [36].

### 3.4. Functionalization of the Nominated Cellulosic Substrates

Functionalization of mill-scoured and bleached cellulosic fabrics was carried out by grafting of monochlorotriazinyl β-cyclodextrin (MCT-βCD), as an environmentally friendly encapsulating and hosting reactive βCD, onto cellulose structure to create core-shaped hydrophobic cavities as follows:


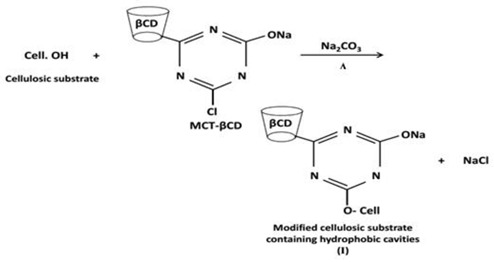


This was followed by subsequent inclusion of the nominated bioactive extracts into the host internal cavities of the grafted reactive βCD via formation of host–guest inclusion complexes [37] as follows:


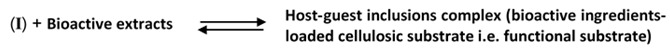


### 3.5. Antimicrobial Properties of the Treated Cellulosic Textiles

The imparted antimicrobial activity of the pre-modified/post-treated substrates with the selected bioactive extracts as potentially eco-friendly alternatives to synthetic antimicrobial agents are presented in Table 3. For a given set of treatment conditions, it is clear that pre-modification of the nominated cellulosic substrates with MCT-βCD had practically no inhibitory effect against the tested bacterial and fungal strains. Inclusion of any of the selected bioactive extracts into the hydrophobic cavities at treated fabrics surface brought about an improvement in their antimicrobial functionality which followed the decreasing order: M113 > 7S4 > 13A >> non-treated. It could be presumed that the extent of improvement in the imparted antimicrobial functionality of bioactive extract-loaded substrates was governed by their concentration, chemical composition, active ingredients, degree of inclusion into, and release out of the hydrophobic cavities at the finished fabrics surface as well as mode and degree of microbial inhibition. On the other hand, the efficacy of the imparted antimicrobial activity, expressed as the size of inhibition zone, against the targeted microorganisms was determined by the type of target microorganism, e.g., bacteria or fungi, their cell wall structure, their amenability to damage, and their capability to inactivate bioactive ingredients present in the bioactive extracts tested. The encapsulated bioactive extracts had practically no (in the case of M113 or 7S4) or only moderate inhibitory effects (as in the case of 13A) against the *E. coli* strain [38,39,40,41,42].

Moreover, the extent of pre-modification and subsequent post-loading and releasing of the nominated bioactive ingredients, which in turn affected the antimicrobial functionality, were governed by type of cellulosic substrates and followed the decreasing order: knitted viscose > knitted cotton > woven cotton fabric, keeping other parameters constant. This reflects the differences among these substrates in weight, thickness, fabric construction amorphous/crystalline area, number, and location of accessible active site, i.e., –OH groups, extent of pre-modification, post-loading, and slow releasing of the active antimicrobial ingredients into the surrounding zone to inhibit the growth or to kill the pathogenic microorganisms [42,43].

### 3.6. Ultraviolet Protection

From Table 4, it can be seen that inclusion of the selected bioactive extracts into the hydrophobic cavities of the modified cellulosic substrates resulted in a remarkable enhancement in their UV protection functionality, expressed as UPF values, regardless of the substrates used and the active ingredients included. According to Table 4, the extent of improvement in UPF values depended on type of substrate, i.e., knitted cotton > woven cotton fabric > knitted viscose fabric, reflecting the differences among them in thickness, porosity, extent of modification, and subsequent hosting of the bioactive extracts ingredients. On the other hand, post-loading of the selected extracts onto the finished fabrics surface to impart UV protection capability was accompanied by a significant increase in their UPF values with the following sequence, 13A > M113 > 7S4 >> modified ≈ untreated. In line with previous studies, we would assume that the variation in UPF values of the finished substrates upon using these ingredients reflects the differences among them in extent of loading and coating the treated substrates as well as the positive role of the hosted bioactive extracts and their ingredients in absorbing, reflecting, scattering, or blocking harmful UV radiation, especially UVA and UVB, irrespective of the substrate used [44,45].

### 3.7. Fungal Extracts Toxicity Study

Because of global concern of functionalized textile toxicity, it was very important to test the selected fungal extracts for their toxicity. Accordingly, the three crude extracts used in textile functionalisation (M113, 7S4, and 13A) were investigated for their cytotoxicity on normal human diploid fibroblasts (WI-38) in comparison with docetaxel as reference control (IC_50_ 22.2 µg/mL). The results obtained demonstrated that M113 extract has a very low toxic effect with an IC_50_ value of 88.6 µg/mL, followed by 13A extract which exhibited weak to moderate toxicity at an IC_50_ value 68.4 µg/mL, while significant toxicity was observed for strain 7S4 with an IC_50_ value 19.3 µg/mL.

### 3.8. Chemical Investigation Using a Molecular Network Approach

**Figure 2:**
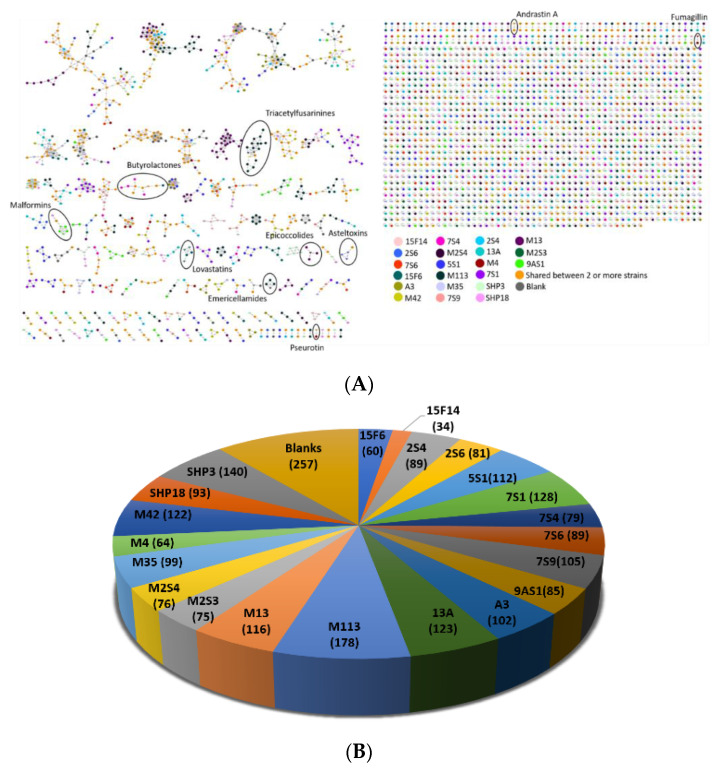
(**A**) Molecular network of the organic extracts of 21 biologically active Egyptian fungal strains. Nodes are colour-coded based on fungal strain ID, as shown in Table 1. The reverse triangle-shaped nodes represent previously reported metabolites identified by Global Natural Products Social Molecular Networking (GNPS)’ libraries. Molecular families are shown in circles. (**B**) Distribution of unique nodes for each fungal strain observed in the molecular network.

The same 21 fungal extracts selected for molecular identification, as described above (Table 1), were subjected for further chemical investigation based on a comparative untargeted metabolomics study using the Global Natural Products Social Molecular Networking (GNPS) platform (https://gnps.ucsd.edu). Analysis of the MS/MS data of the crude extracts was carried out by combining manual dereplication with the aid of various databases such as Natural Products Atlas (NPAtlas, [46] https://www.npatlas.org), AntiBase [47] (https://application.wiley-vch.de/stmdata/antibase.php), and Reaxys (https://www.reaxys.com) as well as automated dereplication using the GNPS platform. The generated molecular network (MN) (Figure 2A) consisted of 2765 nodes in total, out of which 257 corresponded to parent ions present in the media and the solvent blanks, which were excluded from the data analysis. It is worth mentioning that some nodes correspond to different quasi-molecular ions pertaining to the same molecular formula; therefore, not all observed nodes represent a single molecule. The distribution of nodes according to each fungal strain varied across all 21 selected isolates and is given in Figure 2B. The isolates for which the largest number of nodes were observed include *Aspergillus calidoustus* strain M113 (178 nodes) and *Cladosporium spinulosum* strain SHP3 (140 nodes), whereas *Aspergillus fumigatus* strain 15F14 and *Aspergillus terreus* strain 15F6 produced the lowest number of nodes with 34 and 60, respectively. In the MN, different molecular families were observed which correspond to metabolites that are commonly produced by fungi. It is important to take into consideration that manual and automated dereplication cannot provide conclusive results for all 2765 nodes that were present in the molecular network, therefore, herein we report and describe previously known fungal metabolites as well as potentially new derivatives based on the generated molecular network. Additionally, it should be noted that a definite identification of any known compounds by MS/Ms alone is not possible, as it is not possible to deduct the absolute configuration, and the potential presence of isomers cannot ultimately be ruled out. However, from a practical point of view, in our view, the likelihood of the identifications presented in the following being correct would be considered high enough (especially if more than one known compound has been pinpointed within the same cluster, and also taking into account taxonomic information, e.g., previous report from related species) that subsequent isolation efforts would be deemed not worthwhile unless the analysis of the GNPS clusters in question also suggests the presence of potentially unknown compounds.

**Figure 3:**
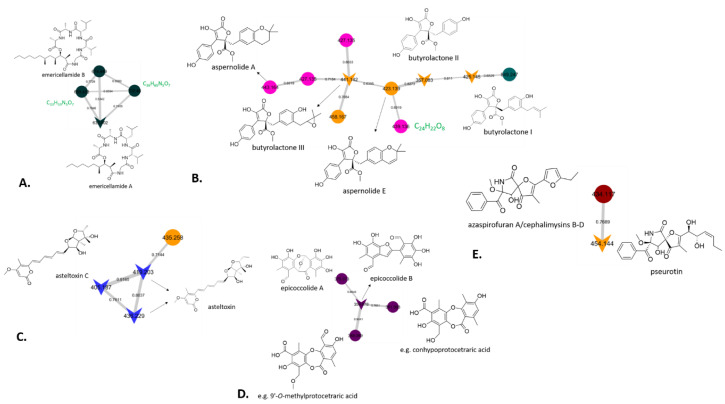
Selected GNPS clusters representing molecular families: (**A**) emericellamide cluster, (**B**) butyrolactone cluster, (**C**) asteltoxin cluster, (**D**) epicoccolide cluster, and (**E**) pseurotin cluster. Nodes depicted by inverted triangular shapes represent hits in the GNPS compound library. Candidate molecular formulae for potential new derivatives highlighted in green.

More specifically, the previously reported cyclic depsipeptide emericellamide A (*m*/*z* 632.402, [M+Na]^+^) was identified by GNPS and was produced by *Aspergillus calidoustus* strain M113, which exhibited the best antimicrobial properties using the treated cellulosic textiles and a low toxicity profile. Manual dereplication allowed the identification of emericellamide B (*m*/*z* 674.449 [M+Na]^+^), which is indeed linked with emericellamide A in the same molecular cluster (Figure 3A). Emericellamides A and B were originally isolated from a marine-derived fungus *Emericella* sp. during co-cultivation experiments with the actinomycete *Salinispora arenicola* [48]. Dereplication with AntiBase of the additional parent ions *m*/*z* 660.433 ([M+Na]^+^) and *m*/*z* 702.480 ([M+Na]^+^) in the emericellamide cluster identified by GNPS provided no hits, which strongly suggests the presence of new emericellamide homologues, with the candidate molecular formulae suggesting an additional two or five methylene groups compared to emericellamide A, respectively. Even though there are reported lipodepsipetides with the same molecular formulae, such as scopularides and oryzamides [49], considering the structures and the relatively high cosine scores in the GNPS cluster, it is very likely that the potentially new derivatives share the same peptide backbone, and that these modifications are located in the polyketide-derived side chain. Taken together, it is highly likely that *Aspergillus calidoustus* strain M113, which was among the most promising in all of our biological assays, may produce undescribed representatives of the emericellamide family of cyclic peptides, warranting future efforts at their targeted isolation and structural characterisation.

Moreover, GNPS identified a cluster containing butyrolactone I (*m*/*z* 425.145, [M+H]^+^), II (*m*/*z* 357.083, [M+H]^+^) and III (*m*/*z* 441.142, [M+H]^+^), which were observed in two strains of *Aspergillus terreus* (7S4, 15F6) and in one isolate of *Penicillium crustosum*, M35 (Figure 3B). Butyrolactones are commonly found in fungal strains and have exhibited a wide range of biological activities [50,51]. Our identifications were based on matching spectra in the GNPS library, although the mass error of the pertaining molecular formulae was relatively high (data not shown). Therefore, manual analysis of the MS/MS of the three fungal extracts was carried out, which confirmed the presence of the butyrolactone derivatives, and, subsequently, butyrolactones I and II were isolated from strain 7S4, and their identity was established by NMR spectroscopy (data not shown). On this basis, while not contained in the GNPS library, further nodes in Figure 3B in all likelihood represent the known aspernolides A [52] and E [53], which are closely related to butyrolactones. It is worth noting that the node with *m*/*z* 439.134, corresponding to the putative molecular formula, C_24_H_22_O_8,_ was only produced by strain 7S4 and gave no hits when it was dereplicated manually, which suggests it may correspond to a new butyrolactone (or aspernolide) derivative. It is worth mentioning that strain 7S4 showed moderate toxicity against normal human diploid fibroblasts, although there is no indication in the literature of the toxic nature of butyrolactones, which warrants further investigation for non-traced metabolites which could be responsible for such toxicity. Additionally, further chemical investigation of this strain would allow the isolation of this potentially new metabolite, which highlights the power of sample prioritisation among large datasets using molecular networking.

Strain 5S1, which, based on the sequencing results should be closely related to *Aspergillus ochraceopetaliformis*, was the only producer of asteltoxin and asteltoxin C which clustered in the same molecular family (Figure 3C). Manual dereplication revealed that a further parent ion in this cluster with *m*/*z* 435.258 ([M+H]^+^) was also observed in the extract of *Alternaria alternata* strain 13A, which exhibited good antibacterial property in the textile assay but was associated with moderate toxicity, which in all likelihood could be attributed to the presence of asteltoxin. While its nominal mass matches that of the known asteltoxin B, the mass error would suggest that the molecular formula may not, thus not fully ruling out the presence of a new asteltoxin derivative, which could only be clarified by further chemical investigation. The mycotoxin asteltoxin was isolated in 1979 from *Aspergillus stellatus* [54], whereas asteltoxin B was obtained from the liquid cultures of an *Aspergillus* sp. strain isolated from the octocoral *Melitodes squamata* [55]. Its structure was later revised in the course of a chemical study of the entomopathogenic fungus *Pochonia bulbillosa*, during which it was obtained along with asteltoxin C [56]. The toxicity associated with strain 13A could be attributed to the presence of such metabolites.

In the epicoccolide cluster, epicoccolide B (*m*/*z* 359.078, [M+H]^+^) was identified by GNPS, whereas epicoccolide A (*m*/*z* 375.073, [M+H]^+^) was confirmed through manual analysis (Figure 3D). Both polyoxygenated polyketides were previously described from an endophytic fungus *Epicoccum* sp. [57]. Interestingly, two further nodes with *m*/*z* 361.093 ([M+H]^+^) and *m*/*z* 389.089 ([M+H]^+^) gave no hits in the GNPS library, while manual dereplication of their candidate molecular formulae, C_18_H_16_O_8_ and C_19_H_16_O_9_, respectively, could not rule out that they may represent known depsidones, for example, conhypoprotocetraric acid [58] and 9′-O-methylprotocetraric acid [59], previously described from terrestrial lichens (besides a large number of other, chemically less closely related compounds with matching molecular formulae). Ultimately, it is not possible to come to a definite conclusion as to whether these compounds are known or may represent new compounds on the basis of LC/MS and GNPS data alone, thus further chemical investigation is required.

Pseurotin was originally isolated in the late 1970s from *Pseudeurotium ovalis* [60] and in the present study was identified in the obtained molecular network as a product of *Aspergillus fumigatus* strain 7S6 and *Talaromyces verruculosis* strain FAS-10 (M4). Pseurotin clustered with the node *m*/*z* 434.117 ([M+Na]^+^) present only in the extract of the latter fungus, and manual dereplication suggested the presence of one out four fungal compounds with a molecular formula of C_22_H_21_NO_7_ (Figure 3E). Azaspirofuran A [61] and cephalimysins B, C, and D [62] are all structurally related to pseurotin, however, they cannot be differentiated in the molecular network because they are stereoisomers, which highlights the limitation of MS/MS data and, therefore, GNPS in identifying isomers. In order to identify which of those four fungal metabolites corresponds to the observed node, chromatographic isolation and structure elucidation are required, but it should be noted that such an effort is not worthwhile, as it will very likely result in the re-isolation of known compounds.

## 4. Discussion

The rates of nosocomial infections, especially by those caused by the newly emerged antibiotic resistant bacteria, are increasing and doing so more alarmingly in the developing countries [63]. The rate of such infections has recently increased, especially in some teaching hospitals in Egypt. As textiles are an excellent substrate for bacterial growth under appropriate moisture and temperature conditions, it is not surprising that several studies have confirmed that personal contact with contaminated textiles is considered the main source of transmission of the microbial infections to susceptible patients, while indirect contact or aerosol transmission of nosocomial pathogens may also be contributing factors [64]. Few studies have addressed the use of antimicrobial textiles, especially for those staff that are in close contact with the patients. A recent study has proven that treating textiles with ionic silver after washing resulted in a significant decrease in microbial contamination, especially *S. aureus* and MRSA contamination [21].

In 2017, we initiated a collaborative project between Egypt and the UK aiming at the isolation of new endophytic fungal strains from under-explored marine habitats in different locations in Egypt to be screened for their antimicrobial effect and the incorporation of the most bioactive extracts in textiles used in Egyptian hospitals to reduce nosocomial infections. We managed to isolate 32 fungal strains from different under-explored marine habitats in Egypt. Based on antimicrobial screening against different microbial pathogens together with the total antioxidant capacity (TAC) of the fungal extracts, 21 endosymbiotic fungal isolates were selected and identified using 18S, ITS, β-tubulin, or calmodulin gene sequencing and were prioritised for further analysis. Further, inclusion of the three most active fungal extracts into the hydrophobic cavities of grafted MCT-βCD onto the cellulosic fabrics surface indicated that *Aspergillus calidoustus* strain M113 exhibited the most promising improvement in the antimicrobial textile functionality and the second best in the UV protection of functionalised cellulosic fabrics while showing only low/weak toxicity against normal human skin fibroblasts. Large scale production of this bioactive extracts along with their industrial application to develop eco-friendly multifunctional cellulosic fabrics will be carried out in our forthcoming study to determine the total cost and the feasibility.

To highlight the possible chemistry behind such antimicrobial and TAC effects, chemical investigation was carried out by the analysis of their LC-MS/MS data through the GNPS platform. The results revealed several already reported antimicrobial compounds which clustered with unidentified parent ions, suggesting the presence of new secondary metabolites. *Aspergillus calidoustus* strain M113 produced previously reported emericellamide derivatives [48] but also two potentially new congeners. The second strain with promising antimicrobial textile functionality and the third for UV protection was *Aspergillus terreus* strain 7S4, which produces a suite of known butyrolactones and aspernolides [49,50,51,52,53] besides one potentially new derivative. However, its skin toxicity precluded further analysis. The third recognised strain for its antimicrobial textile functionality and the best among the best in the UV-protection was *Alternaria alternata* strain 13A, but this was associated with mild skin toxicity, which could be attributed to the presence of asteltoxin based on its GNPS analysis.

## 5. Conclusions

In conclusion, during our Newton funding institutional links project between UK and Egypt, we managed to isolate and characterise different marine-derived endophytes from under-explored habitats in Egypt. Biological screening, textile functionalisation, and GNPS analysis allowed us to prioritise three fungal strains for future in-depth studies due to their promising chemical and biological results, which would lead to the discovery of a convenient approach to reduce the spread of nosocomial pathogens through contaminated textiles in susceptible patients by offering safe and environmentally friendly hospital textiles.

## Figures and Tables

**Table 1:** Origin and identification of fungal strains by DNA sequencing.

Strain ID	Target Gene and GenBank Accession Number				ID Assigned in GenBank	Sample Origin	Type	Location
	18S	ITS	β-Tubulin	Calmodulin				
A3	-	MN114540	MT184332	MT184352	*Aspergillus terreus* strain A3	*Crella cyathophora*	sponge	Makady Bay South, Hurghada
M113	-	MK262919	MT184334	MT184354	*Aspergillus calidoustus* strain M113	*Thalassia hemprichii*	sea grass	Makady Bay South, Hurghada
M13	-	MK953943	MT184348	-	*Epicoccum nigrum* strain FAS-14	*Thalassia hemprichii*	sea grass	Makady Bay South, Hurghada
M2S3	MN328309	MT152324	MT184340	MT184360	*Byssochlamis spectabilis* strain M2S3	*Crella cyathophora*	sponge	Makady Bay South, Hurghada
M2S4	MN328341	MT152319	MT184335	MT184355	*Aspergillus niger* strain M2S4	*Crella cyathophora*	sponge	Makady Bay South, Hurghada
M35	-	MK953944	MT184347	MT184367	*Penicillium crustosum* strain FAS-28	*Siphonochalina siphonella*	sponge	Makady Bay South, Hurghada
M4	-	MK953942	MT184345	MT184365	*Talaromyces verruculosus* strain FAS-10	*Latrunculia magnifica*	sponge	Makady Bay South, Hurghada
M42	MN328356	MT152318	MT184331	MT184351	*Aspergillus terreus* strain M42	*Latrunculia magnifica*	sponge	Makady Bay South, Hurghada
SHP3	-	MN114156	MT184343	MT184363	*Cladosporium spinulosum* strain SHP3	*Thalassia hemprichii*	sea grass	Makady Bay South, Hurghada
SHP18	-	MN114621	MT184342	MT184362	*Cladosporium spinulosum* strain SHP18	*Thalassia hemprichii*	sea grass	Makady Bay South, Hurghada
13A	-	MK248606	-	-	*Alternaria alternata* strain 13A	*Phragmites australis*	plant	Lake El-Bida, Wadi El-Natrun
15F6	-	MN328763	MT184330	MT184350	*Aspergillus terreus* strain 15F6	*Hyoscyamus muticus*	plant	Lake El-Bida, Wadi El-Natrun
15F14	MN328048	MT152320	MT184336	MT184356	*Aspergillus fumigatus* strain 15F14	*Hyoscyamus muticus*	plant	Lake El-Bida, Wadi El-Natrun
2S4	-	MN115554	MT184341	MT184361	*Alternaria alternata* strain 2S4	*Juncus acutus*	plant	Lake El-Hamra, Wadi El-Natrun
2S6	-	MN330611	MT184338	MT184358	*Aspergillus fumigatus* strain 2S6	*Juncus acutus*	plant	Lake El-Hamra Wadi El-Natrun
5S1	-	MN110110	MT184339	MT184359	*Aspergillus ochraceopetaliformis* strain 5S1	*Panicum turgidum*	plant	Lake El-Hamra, Wadi El-Natrun
7S1	-	MK953941	MT184346	MT184366	*Talaromyces verruculosus* strain FAS-02	*Tamarix nilotica*	plant	Lake El-Hamra, Wadi El-Natrun
7S4	-	MN114521	MT184333	MT184353	*Aspergillus terreus* strain 7S4	*Tamarix nilotica*	plant	Lake El-Hamra, Wadi El-Natrun
7S6	MN326853	MT152321	MT184337	MT184357	*Aspergillus fumigatus* strain 7S6	*Tamarix nilotica*	plant	Lake El-Hamra, Wadi El-Natrun
7S9	-	MN114217	MT184344	MT184364	*Penicillium rubens* strain 7S9	*Tamarix nilotica*	plant	Lake El-Hamra, Wadi El-Natrun
9AS1	MN327968	MT152323	MT184349	MT184368	*Sarocladium kiliense* strain 9AS1	*Panicum turgidum*	plant	Lake El-Hamra, Wadi El-Natrun

**Table 2:** Antimicrobial activity of the fungal extracts.

Sample Code	*Staphylococcus aureus* MIC (µg/mL) *	*Pseudomonas aeruginosa* MIC (µg/mL) *	*Candida albicans* MIC (µg/mL) *	*Aspergillus niger* MIC (µg/mL) *
M1 °	250	250	250	-
M113	25.0 ± 1.7	22.3 ± 1.5	52.3 ± 0.6	192.7 ± 3.8
M13	20.0 ± 2.0	42.0 ± 1.0	111.0 ± 1.7	122.7 ± 4.6
M23 °	250	250	250	-
M2S3	100.3 ± 4.0	195.0 ± 6.2	177.3 ± 4.5	-
M2S4	115.0 ± 2.6	102.7 ± 3.8	163.3 ± 2.3	-
M32 °	250	250	250	-
M35	85.0 ± 2.6	101.0 ± 1.7	180.7 ± 5.0	237.7 ± 2.1
M4	172.3 ± 4.0	185.0 ± 3.6	158.7 ± 4.7	210.3 ± 4.2
M42	43.0 ± 1.0	86.0 ± 2.6	92.7 ± 3.8	-
SHP3 °	-	-	35.0 ± 1.7	-
SHP18	105.0 ± 5.6	123.7 ± 0.6	203.0 ± 2.6	-
13A	45.0 ± 1.0	42.0 ± 1.0	102.7 ± 3.1	-
2S4	48.0 ± 2.6	47.3 ± 3.2	87.7 ± 3.8	85.0 ± 4.4
2S6	97.7 ± 2.1	103.7 ± 2.9	209.0 ± 2.0	-
5S1	50.0 ± 2.6	46.3 ± 3.5	85.3 ± 3.2	125.0 ± 0.0
7S1	54.0 ± 1.7	51.0 ± 2.0	102.7 ± 2.9	183.7 ± 5.0
7S4	12.3 ± 1.5	13.7 ± 1.2	52.3 ± 1.5	-
7S5 °	31.25	62.5	125	-
7S6	113.7 ± 0.6	55.7 ± 1.2	175.0 ± 2.6	-
7S9	47.3 ± 3.2	41.0 ± 2.6	171.0 ± 1.5	-
9AS1	117.7 ± 4.2	43.7 ± 3.1	-	-
Cip	0.078	0.156	-	-
Nys	-	-	5	10

* Based on three independent replicates; ° only tested in the initial serial dilution, see Experimental section. Cip: ciprofloxacin; Nys: nystatin; -: not active; MIC: minimum inhibitory concentration.

**Table 3:** Antimicrobial properties of the treated cellulosic textiles.

Extract (Concentration Tested)	Fabric Type ^a^	Zone of Inhibition (mm)			
		*S. aureus*	*E. coli*	*C. albicans*	*A. niger*
M113 (55 µg/mL)	1	16	-	17	15
	2	18	-	18	15
	3	19	-	19	17
7S4 (70 µg/mL)	1	13	-	14	11
	2	14	-	15	12
	3	16	-	16	14
13A (62 µg/mL)	1	12	7	11	10
	2	13	8	12	11
	3	14	9	13	12
modified	1	-	-	-	-
	2	-	-	-	-
	3	-	-	-	-
untreated	1	-	-	-	-
	2	-	-	-	-
	3	-	-	-	-

^a^ 1; woven; 2, C. knitted; 3, V. knitted.

**Table 4:** UV protective properties of different textile fabrics treated with selected fungal extracts.

Extract (Concentration Tested)	Fabric Type ^a^	UPF	Classification
M113 (55 µg/mL)	1	70.8	excellent
	2	162.2	excellent
	3	30.7	Very good
7S4 (70 µg/mL)	1	33.0	very good
	2	77.6	excellent
	3	26.9	very good
13A (62 µg/mL)	1	113.9	excellent
	2	202.8	excellent
	3	38.3	very good
modified	1	10.5	no protection
	2	15.8	good
	3	8.0	no protection
untreated	1	7.0	no protection
	2	11.2	no protection
	3	5.0	no protection

^a^ 1; woven; 2, C. knitted; 3, V. knitted. UPF: UV protection factor.
